# Targeting immunotherapy for bladder cancer by using anti-CD3 × CD155 bispecific antibody

**DOI:** 10.7150/jca.29937

**Published:** 2019-08-28

**Authors:** Wanru Ma, Juan Ma, Ting Lei, Man Zhao, Man Zhang

**Affiliations:** 1 Department of Clinical Laboratory Medicine, Beijing Shijitan Hospital, Capital Medical University, Beijing, China; 2 Peking University Ninth School of Clinical Medical, Beijing, China; 3 Beijing Key Laboratory of Urinary Cellular Molecular Diagnostics, Beijing, China; 4 Collage of Medical Technique, Xuzhou Medical University, Jiangsu, China

**Keywords:** bladder cancer, immunotherapy, CD155, bispecific antibody

## Abstract

To investigate whether CD155 is an attractive target for T cell-mediated immunotherapy against human bladder cancer, we examined the novel bispecific antibody anti-CD3 x anti-CD155 (CD155Bi-Ab) for its ability to redirect activated T cells (ATCs) to target bladder cancer cells was examined. Expression of CD155 was detected by flow cytometry on the surface of bladder cancer cells, including T24 and Pumc-91 cells, and their chemotherapeutic drug-resistant counterparts. ATCs generated from healthy donors were stimulated with anti-CD3 monoclonal antibody, anti-CD28 monoclonal antibody and interleukin-2 (IL-2) for 14 days. The cytotoxic activity of ATCs armed with CD155Bi-Ab against bladder cancer cells was detected by LDH and luciferase quantitative assay. Furthermore, ATCs generated from bladder cancer patients were also armed with CD155Bi-Ab to verity the cell killing by the same methods. In contrast to unarmed ATCs, CD155Bi-armed ATCs against bladder cancer cells were increased cytotoxic activity at effector/target (E/T) ratios of 5:1, 10:1, and 20:1, with more IFN-γ, TNF-α secreting. It is worth noting that in spite of the presence of immunosuppression in bladder cancer patients and the drug resistance in chemotherapeutic drug-resistant cancer cell lines, not only the anti-tumor effect of CD155Bi-armed ATCs generated from bladder cancer patients still showed significantly but only higher level of activation marker CD69 was expressed. Taken together, our results suggest that CD155 is an effective target for the CD155-positive bladder cancer. And CD155Bi-Ab-armed ATCs are promisingly to provide a novel strategy for current CD155-positive bladder cancer therapy.

## Introduction

Bladder cancer is one of the most common cancers among people, which ranks 9th of world cancer and caused 165,100 deaths each year, with male morbidity and mortality 4 times higher than female 1,2. Non-muscle invasive bladder cancer accounts for approximately 75% of bladder cancer cases and 40% of these patients will progress to muscle invasive with an average of 15 months survival 3,4. Despite a variety of treatment methods, such as surgery, radiotherapy and chemotherapy, patients with bladder cancer suffer from significant treatment failure, including high rates of recurrence and poor outcomes for advanced disease 5. Therefore, there is an urgent need for a new treatment for bladder cancer.

Researchers are optimistic that immunotherapy is a major breakthrough in treating cancer patients 6. Cancer immunotherapies including “passive” and “active” methods. Passive immunotherapeutic methods involving of immune responses induction through transfer of tumor antigen-targeted monoclonal antibodies and the activation of endogenous immunity by T cells 7. Active immunotherapeutic methods including cancer vaccines, bispecific antibodies (Bi-Abs) to induce cancer immunity 8.

CD155, a membrane protein which belongs to the immunoglobulin (Ig) superfamily, has been confirmed to overexpress in several kinds of human malignancies, including lung adenocarcinoma, melanoma, pancreatic cancer and colon cancer 9-14. CD155 is associated with tumor progression, knockdown of CD155 can inhibit the invasion and migration of glioma cells 15-16. CD155 down-regulation also inhibited colon cancer cell proliferation, accompanied by the inhibition of colon cancer cell migration and invasion 17. In spite of the role of CD155 in bladder cancer remains unknown, all of the above indicate that CD155 may become an immunotherapeutic target for bladder cancer.

Against the above background, we demonstrated the high-level expression of CD155 on human bladder cancer cells. Anti-CD3 antibody was chemically conjugated with anti-CD155 antibody to synthesis the anti-CD3 × anti-CD155 bispecific antibody (CD155Bi-Ab), with anti-CD155 antibody recognized the bladder cancer cells and anti-CD3 antibody recognized the T cells. Activated T cells (ATCs) which from both healthy donors and bladder cancer patients were armed with CD155Bi-Ab to kill bladder cancer cells.

## Materials and Methods

### Cell line

The human bladder cancer pumc-91 cell line was provided by the Cell Laboratory of Beijing Union Medical College Hospital. The drug-resistant bladder cancer cell line pumc-91/ADM was established by adding the dosage of Adriamycin (Aladdin) gradually and the final concentration of Adriamycin was 1.0 ug/ml 18-21. The human bladder cancer T24 cell line was provided by the Chinese Academy of Sciences. The drug-resistant bladder cancer cell line T24/DDP was established by stepwise exposure of T24 cells to escalating concentrations of cisplatin (Sigma-Aldrich, St. Louis, MO, USA). The final concentration of cisplatin was 0.6μg / ml 21-22. The parental cell lines were cultured in RPMI-1640 medium supplemented with 15% fetal bovine serum, while drug-resistant cell lines were cultured in the above-mentioned medium with 18% fetal bovine serum and incubated in an incubator containing 5% carbon dioxide at 37 ° C.

### Isolation of peripheral blood lymphocytes (PBMCs) and preparation and cryopreservation of activated T lymphocytes

Peripheral blood mononuclear cells (PBMCs) were separated immediately by Ficoll-Hypaque density gradient centrifugation. Blood was obtained from healthy donors which supplied by the Beijing Blood Bank. PBMCs were cultured at 1×10^6^/ ml in RPMI-1640 medium supplemented with 10% FBS and 5 μg/ml anti-CD3 mAb (eBioscience, San Diego, CA, USA) combined with 5 μg/ml anti-CD28 mAb (eBioscience) in the presence of 100 IU/ml recombinant human IL-2 (Peprotech, Rocky Hill, NJ, USA). IL-2 was added every 2 or 3 days to stimulate ATC cells and the cells were cultured for 14 days. On day 14, ATC expansion products of healthy donors were on 98.99% CD3+ cells (2.66% CD3+CD4+ cells, 85.95% CD3+CD8+ T cells, and 16.16% CD3+CD56+T cells), the cells were used immediately or cryopreserved for further use.

### Synthesis of anti-CD3 × anti-CD155 Bispecific Antibody (CD155Bi-Ab) and arming of ATCs

1mg/ml of Anti-CD155 mAb (R&D System, Minneapolis, MN, USA) was reacted with 10-fold molar excess of sulfo-SMCC (2 mg/ml) and 1mg/ml of Anti-CD3 (OKT3, eBioscience, San Diego, CA, USA) was reacted with 10-fold molar excess of Traut's reagent (2mg/ml). Both reaction mixtures were incubated for 1 hour at room temperature and removed excess crosslinker using PD-10 column. The anti-CD155-SMCC and anti-CD3-SH were mixed immediately at equimolar ratios and hetero conjugated at 4°C overnight. The products of the hetero conjugation were resolved by SDS-PAGE and stained with Gel code Blue. The concentration was detected by the BCA kit. The coupling rate was calculated by gray scale analysis of western blot using ImageJ software. Cryopreserved ATCs were thawed and armed with CD155Bi-Ab at a concentration of 100 ng/10^6^ cells at room temperature for 30 minutes followed by washing the cells to eliminate unbound antibodies with the combination of OKT3 (100 ng/10^6^ cells) and Anti-CD155-mAb (100ng/10^6^ cells) pre-incubated ATCs using as unarmed control ATCs.

### Flow cytometric analysis

Bladder cancer cells were staining with anti-human CD155-PE mAb and mouse IgG1-PE isotype antibody (eBioscience) to detect the expression of CD155. To verify the successful coupling of CD155Bi-Ab, PUMC-91/ADM cells and ATCs were incubated with CD155Bi-Ab for 30 minutes, and then stained with anti-mouse IgG1-PE or anti-mouse IgG2a-PE. The combination of CD155 with OKT3 was used as the negative control. ATCs were incubated with anti-human CD3-FITC, anti-human CD4-PE, anti-human CD8-APC, and anti-human CD56-APC to examine the population of effector cells. To detect the expression of CD69 on ATCs, floating cells from bladder cancer cells and ATC co-cultures were incubated with anti-human CD69-PE and anti-human CD3-FITC. The anti-human CD3-FITC, anti-human CD69-PE, anti-human CD4-PE, anti-human CD8-APC, anti-human CD56-APC, anti-mouse IgG1-PE and anti-mouse IgG2a-PE secondary antibodies were from eBioscience. The cells were analyzed by the flow cytometer (CytoFlex, Beckman coulter) and data were processed using the accompanying software (CytExpert, Beckman coulter).

### Cytotoxicity assay

Target cells (T24, T24/DDP, PUMC-91, PUMC-91/ADM) were seeded in triplicate in 96-well u bottom microplates at 1x10^4^/well prior to the addition of Anti-CD155Bi-Ab, unarmed ATC or ATC cells at an effector-to-target (E/T) ratio of 10:1. Effector cells and tumor cells were then interacted at 37°C for 18 hours. Cell free supernatants were collected and cytotoxicity was measured by Lactate dehydrogenase activity kit (Sigma-Aldrich, St.Louis, MO, USA).T24-LUC and T24/DDP-LUC were used as target cells to repeat the above experiment at an effector-to-target (E/T) ratio of 1:1,5:1and 20:1.Luciferase quantitative assay 23-27 in which bioluminescence imaging signal in tumor cells expressed in photons per second was converted into living cell number, and the cytotoxicity assays were calculated.

### ELISA assay

The cell free supernatants were collected as described above, and the IFN-γ, TNF-α productions were detected by the human cytokine ELISA kits (eBioscience) according to the manufacturer's instructions.

### Cytotoxicity of ATC from bladder cancer patients

Peripheral blood mononuclear cells (PBMCs) from bladder cancer patients were separated immediately by Ficoll-Hypaque density gradient centrifugation. ATCs were cultured and armed CD155Bi-Ab in the same manner as described above. The killing effect was proved by cytotoxicity assay, ELISA assay and Flow cytometric analysis as described above.

### Statistical analyses

All experiments were repeated at least twice and mostly three times. Data were analyzed by Graphpad Prism 6 software and presented as the means ± SD. Unpaired Student's t-test (two-tailed) was used for comparison of two groups. p<0.05 was considered statistically significant. *P<0.05, **P < 0.01, ***P < 0.001, ****P<0.0001.

## Results

### Verify of CD155 expression on human bladder cancer cells

**Figure 1:**
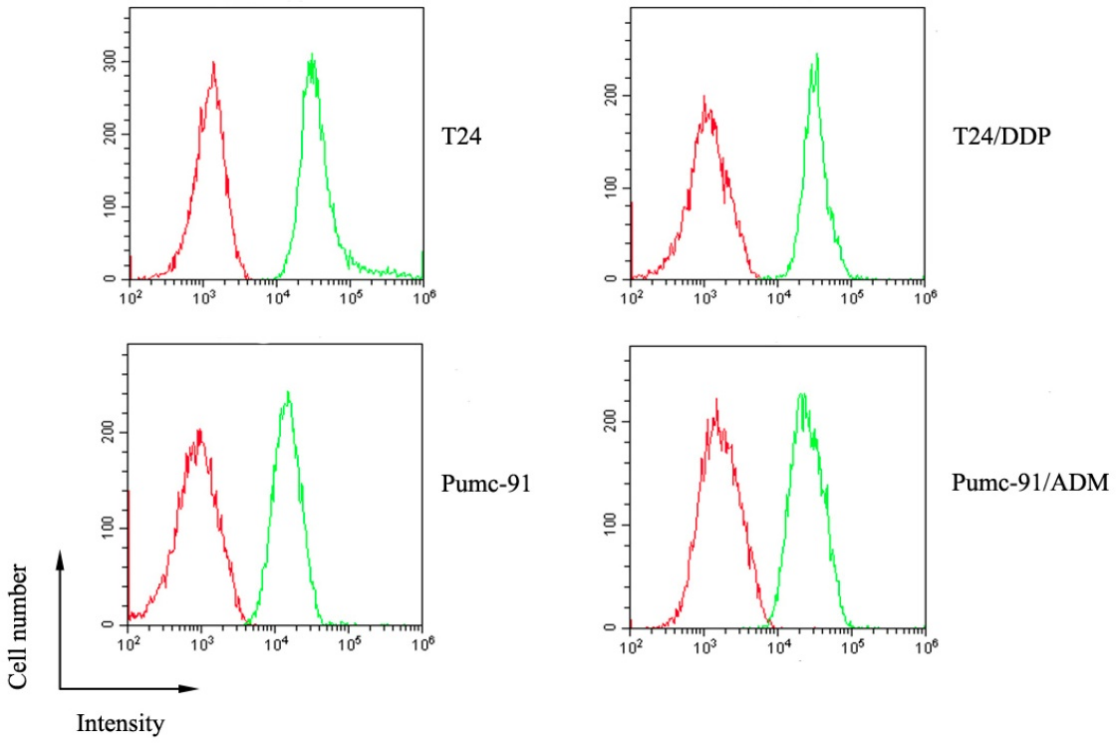
The overexpression of CD155 on human bladder cancer cells (T24, T24/DDP, Pumc-91, Pumc-91/ADM). Green histogram represents cells stained with anti-CD155 mAb and red histogram represents cells stained with the control mouse IgG1.

The expression of CD155 on human bladder tumor cells which includes T24 and Pumc-91cells, and their chemotherapeutic drug-resistant counterparts, T24/DDP and Pumc-91/ADM was evaluated by FACS analysis. As Figure 1 shows, high expression of CD155 was detected on the surface of human bladder cancer cells.

### Characterization of CD155Bi-Ab and preparation of ATCs

The bispecific antibody CD155Bi-Ab which could recognize both CD3 on T cells and CD155 on bladder cancer cells was hetero-conjugated by Anti-human CD155 mAb and OKT3. The equimolar concentrations of binding product were detected by Coomassie blue. The coupling rate which calculated by ImageJ software is 17%. Pmuc-91/ADM and ATC were stained with CD155Bi-Ab or a combination of OKT3 and anti-human CD155 mAb. Anti-mouse IgG2a-PE was identificated the CD3 moiety of CD155Bi-Ab and anti-mouse IgG1-PE was identificated the CD155 moiety of CD155Bi-Ab.

**Figure 2:**
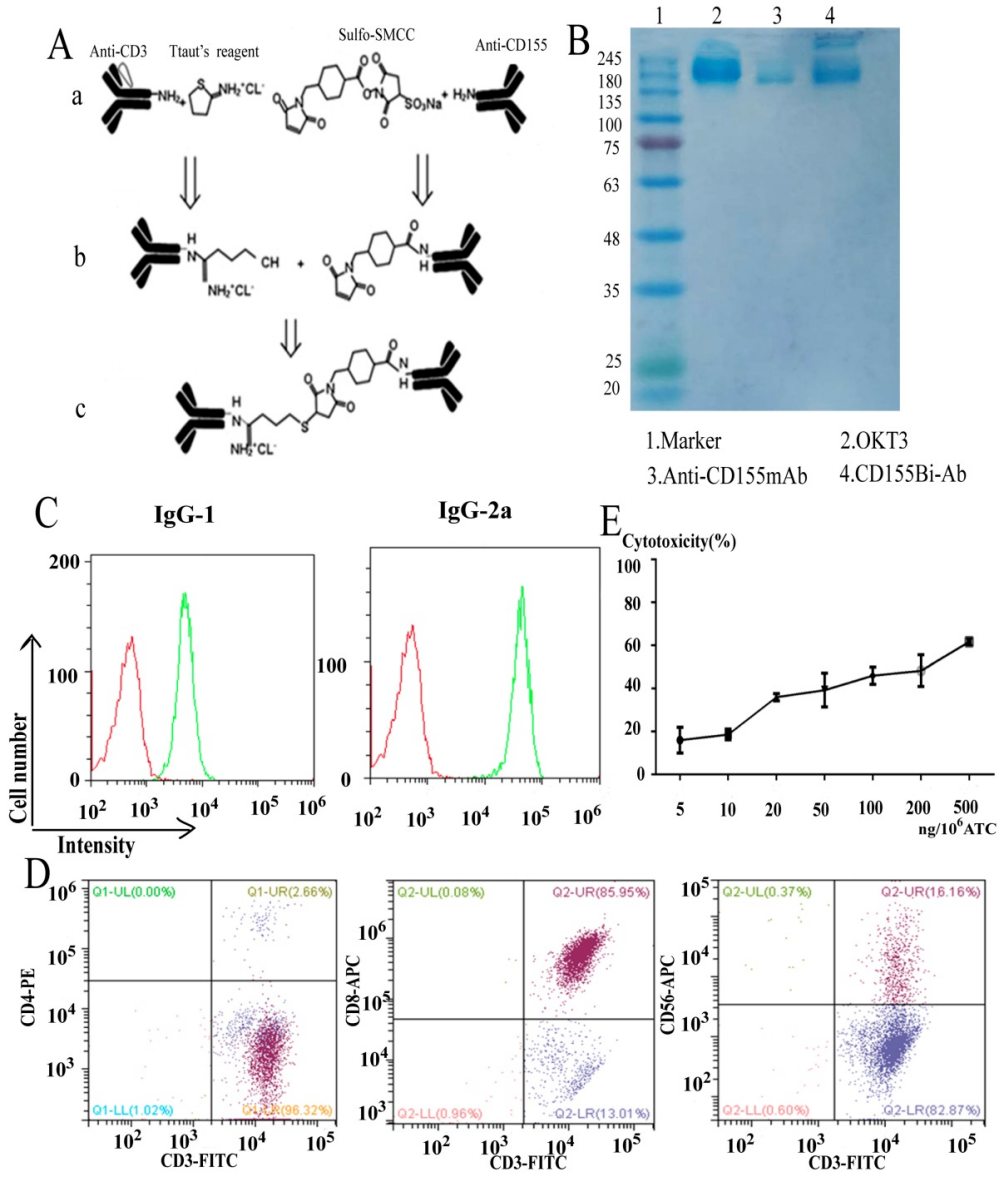
General program for CD155Bi-Ab and analyze of ATC. (A) General program for the generation of CD155Bi-Ab. (B) The concentrations of CD155Bi-Ab were measured by Coomassie blue staining of SDS-gel. (C) The assay of CD155Bi-Ab by flow cytometry. (D) The molecule expression on ATC analyzed by flow cytometry. (E) The optimal concentration of CD155Bi-Ab armed ATC was measured. ATC was armed with CD155Bi-Ab ranging from 5 to 500 ng/10^6^ cells at E/T ratio of 10:1, and the cytotoxic effects of CD155Bi-armed ATC on T24/DDP-luc cells were detected. After one-night incubation of CD155 Bi-armed ATCs with T24/DDP-luc cells, the percentage of viability was calculated at different concentrations.

For the purpose of considerable effective ATCs, PBMCs isolated from peripheral blood of healthy people were stimulated with anti-CD3 mAb and anti-CD28mAb.IL-2 was added to the media every 2 days. On day 14, the molecular expressions of ATCs were detected by FACS analysis. As Figure 2C shows, the cells contained about 99% CD3+ cells, in which there were 85.95% CD3+CD8+ ,2.66% CD3+CD4+ and 16.16% CD3+CD56+ cells. In a word, the above data illustrate that cells mainly consist of ATCs and a small part of NK cells.

To determine the optimal concentration of CD155Bi-Ab, ATC was armed with CD155Bi-Ab ranging from 5 to 500 ng/10^6^ cells, and cytotoxic effects of CD155Bi-armed ATC on T24/DDP-luc cells were detected. After one-night incubation of CD155 Bi-armed ATCs with T24/DDP-luc cells, the percentage of viability was almost 46% at the concentration of 100 ng CD155Bi-Ab/10^6^ cells at effector-to-target (E/T) ratio of 10:1.

### Cytotoxity effects of CD155Bi-armed ATC on bladder tumor cell lines

**Figure 3:**
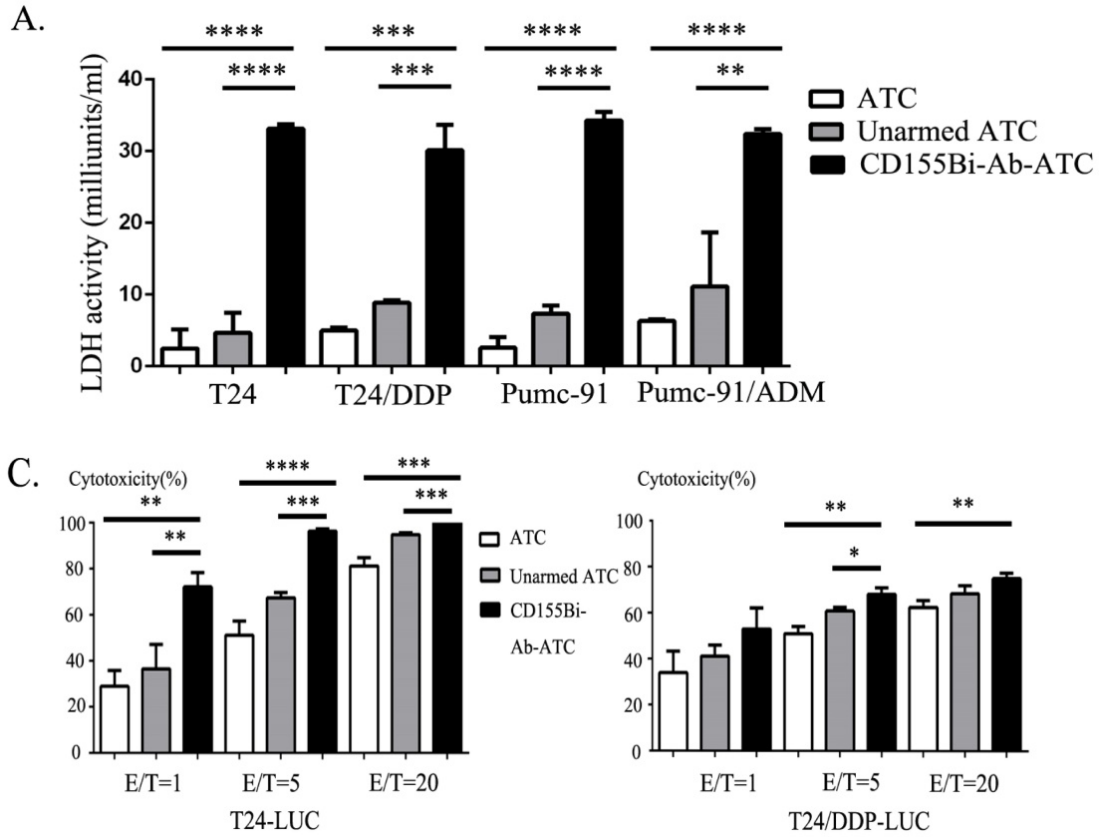

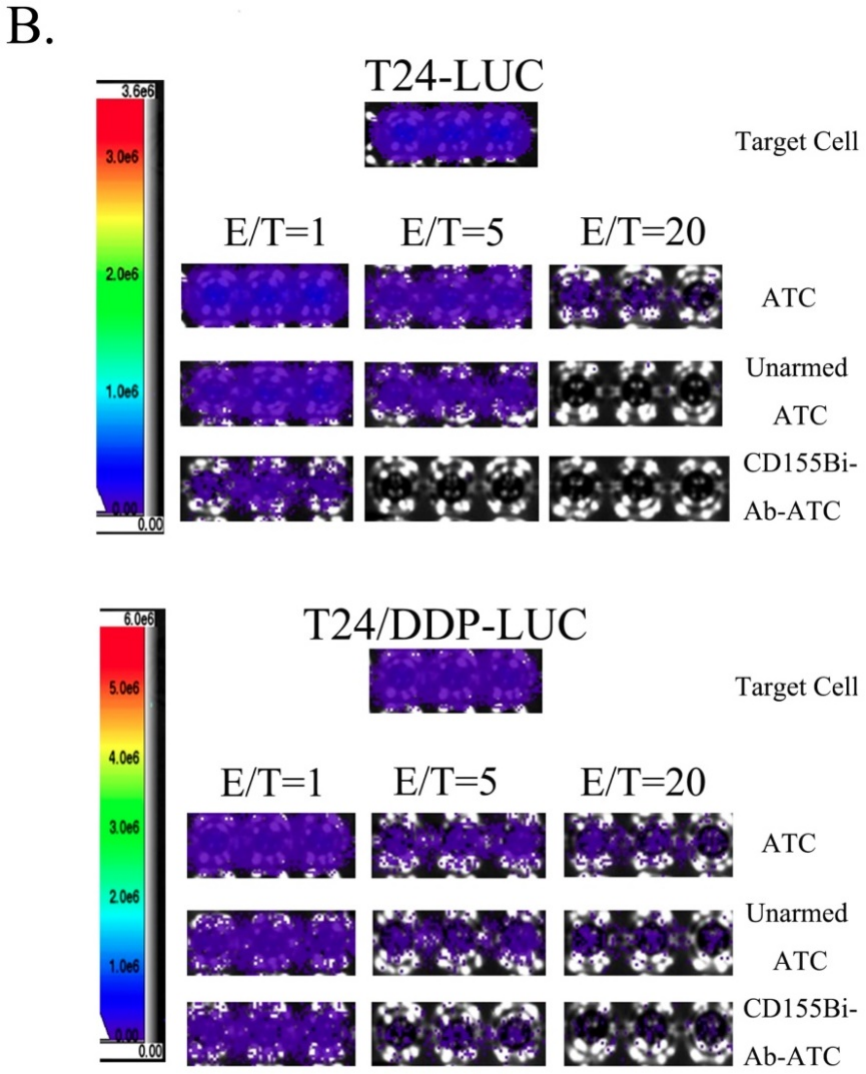
Cytotoxity effects of CD155Bi-Ab-armed ATC on human bladder cancer cells. (A) Supernatants were harvested and analyzed for cytokine levels by lactate dehydrogenase kit. (B) Bioluminescence images of T24-luc cells and T24/DDP-luc cells cultured with CD155Bi-Ab-ATC or unarmed ATC cells at the E/T ratio of 1:1, 5:1 and 20:1. (C). Cytotoxicity assays were calculated.

On account of the concentration of 100ng/10^6^ ATC has shown great kill effect, it was chose as the concentration of CD155Bi-Ab for all the subsequent experiments. ATC mixed with OKT3 and anti-CD155 mAb were used as unarmed ATC controls. Effect cells and target cells were interacted at a ratio of 10:1 at 37 ° C for one night. Real-time photographs of each bladder cancer group were taken at × 200 magnification. The supernatants of co-cultures were collected, with lactate dehydrogenase activity kit used to determine the cytotoxicity. Moreover, effect cells were interacted with stable bladder cancer cell line T24 and cisplatin resistant cell line T24/DDP that expressed luciferase reporter at different ratios (1:1, 5:1and 20:1) and the cytotoxicity were calculated. As Figure 3C shows, fluorescence in CD155Bi-Ab-ATC cell was less than unarmed ATC counterparts, indicating CD155Bi-Ab-ATC has efficacious killing effects on bladder cancer cells.

### Cytokine production by CD155Bi-Ab-ATC on human bladder cancer cells

For the purpose of analyze cytokines generating from ATCs, supernatants were collected for the analyses of IFN-γ, TNF-α productions at E/T ratio of 10:1. Obviously, CD155Bi-Ab-ATCs secret higher level of IFN-γ, TNF-α than other control groups.

### Cytotoxicity of ATC from bladder cancer patients

**Figure 5:**
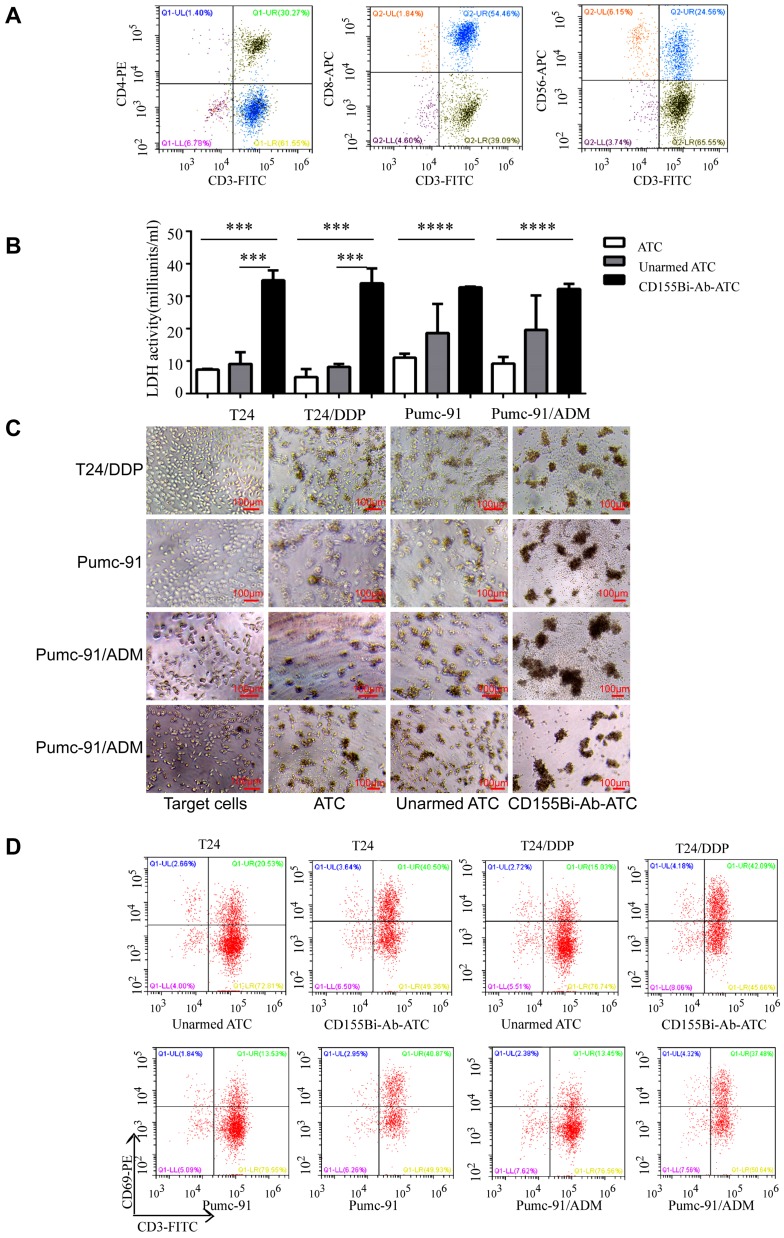
Cytoxicity of ATC from bladder cancer patients. ATC isolated from the peripheral blood of bladder cancer patients were armed with CD155Bi-Ab.Target bladder cancer cells were incubated either with CD155Bi-Ab-ATC or unarmed ATC at E/T ratio of 10:1. (A) The molecule expression on ATC which isolated from the peripheral blood of bladder cancer patients analyzed by flow cytometry. (B) Supernatants were collected after one night and analyzed for LDH activity. (C)Real-time photographs of each bladder cancer group were taken at ×200 magnification. (D) Expressions of CD69 on CD155Bi-Ab-ATC or unarmed ATC were detected by flow cytometry after co-culture with bladder cancer cells.

To confirm whether CD155Bi-Ab can effectively target the patients with bladder cancer, ATC isolated from the peripheral blood of bladder cancer patients were armed with CD155Bi-Ab and the above experiment was repeated. As Figure 5A shows, the cells contained about 92% CD3+ cells, in which there were 54.46% CD3+CD8+ ,30.27% CD3+CD4+ and 24.56% CD3+CD56+ cells. Real-time photographs of each bladder cancer group which armed with ATC, unarmed ATC and CD155Bi-Ab ATC respectively were taken at × 200 magnification (Figure 5B). Significant increases were detected in LDH activity (Figure 5C) secretion in CD155Bi-Ab-ATC compared with their unarmed ATC counterpart when ATC were co-cultured with T24, T24/DDP, Pumc-91, Pumc-91/ADM. Increased CD69 expression on CD155Bi-Ab-ATC over their unarmed ATC after incubation with bladder cancer cells were detected by FACS (Figure 5D). CD69, an early activation marker of lymphocytes, plays the significant role in immune responses.

## Discussion

Bladder cancer is one of the common malignant tumors of the urinary system, with multidrug resistance existed as a serious problem. Due to its safety and efficacy, immunity therapy is recognized as the fourth treatment in tumor comprehensive therapy strategy in the twenty-first century 28, especially the application of BiAbs. BiAbs are a bridge that links the immune lymphocyte and cancer cells, inspiring achievements in tumor immunotherapy.

CD155, an immunoglobulin-like adhesion molecule, has been proved upregulated in a series of human malignancies, such as colon cancer, lung adenocarcinoma, melanoma, pancreatic cancer and glioblastoma 12, 29-32. Research has shown that CD155 plays critical roles in tumor cell invasion and migration 32-34. In reviewing the literature, no date was found on the association between CD155 and bladder cancer. To our knowledge, our study represents the first to examine the intersection of CD155 and bladder cancer.

In this study, we found CD155 is highly expressed on human bladder cancer cells and their chemotherapeutic drug-resistant counterparts. We further tested whether CD155 was an effective target for the immunity therapy in bladder cancer, and evaluated antitumor effects of CD155Bi-Ab-armed ATCs. The results of this study show that compared with control unarmed ATCs, CD155Bi-Ab-armed ATCs secreted more IFN-γ and TNF-α. These increased cytokines generated by activated T cells mediated antitumor activity by activating endogenous immune cells *in vivo* and participated in immune regulation to induce local or systemic immune responses to tumors 35. Microscope images clearly showed CD155Bi-Ab-armed ATCs are activated and gathered around target cells. The supernatants CD155Bi-Ab-armed ATCs cultured with target cells were detected more LDH than control groups. Another method for verifying the killing effect is bioluminescent image that clearly showed fluorescence in CD155Bi-Ab-ATCs was less than that in their unarmed ATC counterparts. The above results showed that T-cell cytotoxicity was played on the engagement of CD155 via Bi-Ab linkage.

For this study the ATCs from bladder patients were used to test and verify the strong killing effect of CD155Bi-Ab. In addition, increased CD69 expression on CD155Bi-Ab-ATCs over their unarmed ATC counterparts after co-culture with bladder cancer cells were detected by FACS analysis.CD69, an early activation marker of T-cell, is regarded as a target for the treatment of immune-mediated diseases and crucial for immune responses 36. The presence of T cells in bladder cancer patients failed to mediate the effect of effective killing of bladder cancer cells. We speculate that because T cells failed to find a suitable “pathway” to target bladder cancer cells. In this study, we provided an effective way of CD155Bi-Ab for T cells to target bladder cancer cells, thereby exerting a powerful role for T cells in killing bladder cancer cells. The above findings make it easier for clinical bladder cancer patients to use the immunotherapy of CD155Bi-Ab.

Our data lend additional support to the CD155 is an immunotherapy target for CD155-positive bladder cancer. Next we will further conduct animal experiments for *in vivo* studies and preclinical studies to verify the antitumor activity of CD155Bi-Ab.

This study has shown that CD155Bi-Ab-ATC can effectively kill CD155-positive bladder cancer cells. Our data generate new hypotheses that CD155 appear to be the target of immunotherapy for the CD155-positive bladder cancer, CD155Bi-Ab-armed ATCs would provide a novel strategy for the immunotherapy of CD155-positive bladder cancer in the future.

## Figures and Tables

**Figure 4:**
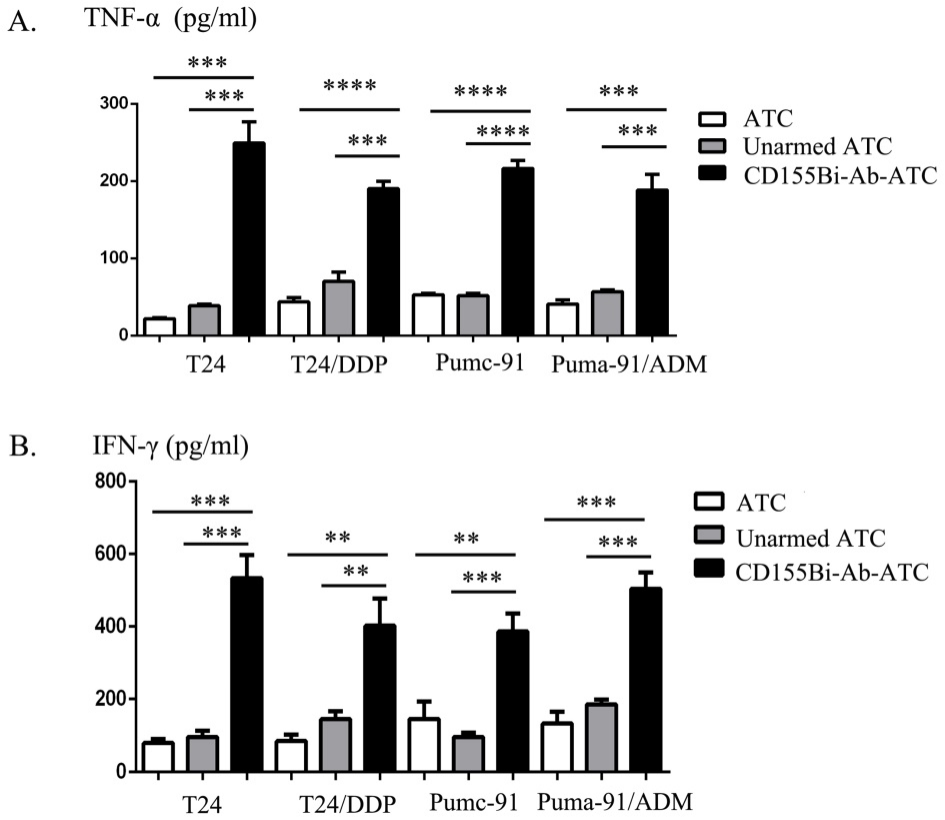
The secretion of TNF-α(A), IFN-γ (B) by CD155Bi-Ab-ATCs against human bladder cancer cells. Supernatants of co-cultures were collected as previously described and analyzed by ELISA kits.
